# Something Novel, Simple Economical and Complete

**Published:** 1882-01-20

**Authors:** 


SOMETHING NOVEL, SIMPLE, ECONOMICAL AND COMPLETE.—The "Physicians' Day-Book and Cash Record, Ledger and Obstetrical Record" combines more elements of ease, beauty and perfection, together with simple accuracy for physicians' book-keeping, than any system hitherto offered in our State. We have examined them and can heartily recommend the books to the brotherhood, as they are suited to all kinds of practitioners. Captain M. E. Cooper is the exclusive and general agent for this State, and all orders from Georgia are filled through him alone. His address is Atlanta. The system adopted is protected by copy-right, and full notes of explanation and specimens accompany the books. P.

